# Supplementary material for: Phycocyanin attenuates skeletal muscle damage and fatigue via modulation of Nrf2 and IRS-1/AKT/mTOR pathway in exercise-induced oxidative stress in rats
Source: PLoS One. 2024 Sep 10;19(9):e0310138. doi: 10.1371/journal.pone.0310138 (PMC11386417; doi:10.1371/journal.pone.0310138)

Supporting information

S1 Raw images of the original western blot images on the PVDF membrane (Merck, MA, USA), staining with Immobilon Forte Western HRP substrate (WBLUF0100 Merck Millipore, MA, USA) and detected by Amersham TM Image Quant 800 (Cytiva, MA, USA).

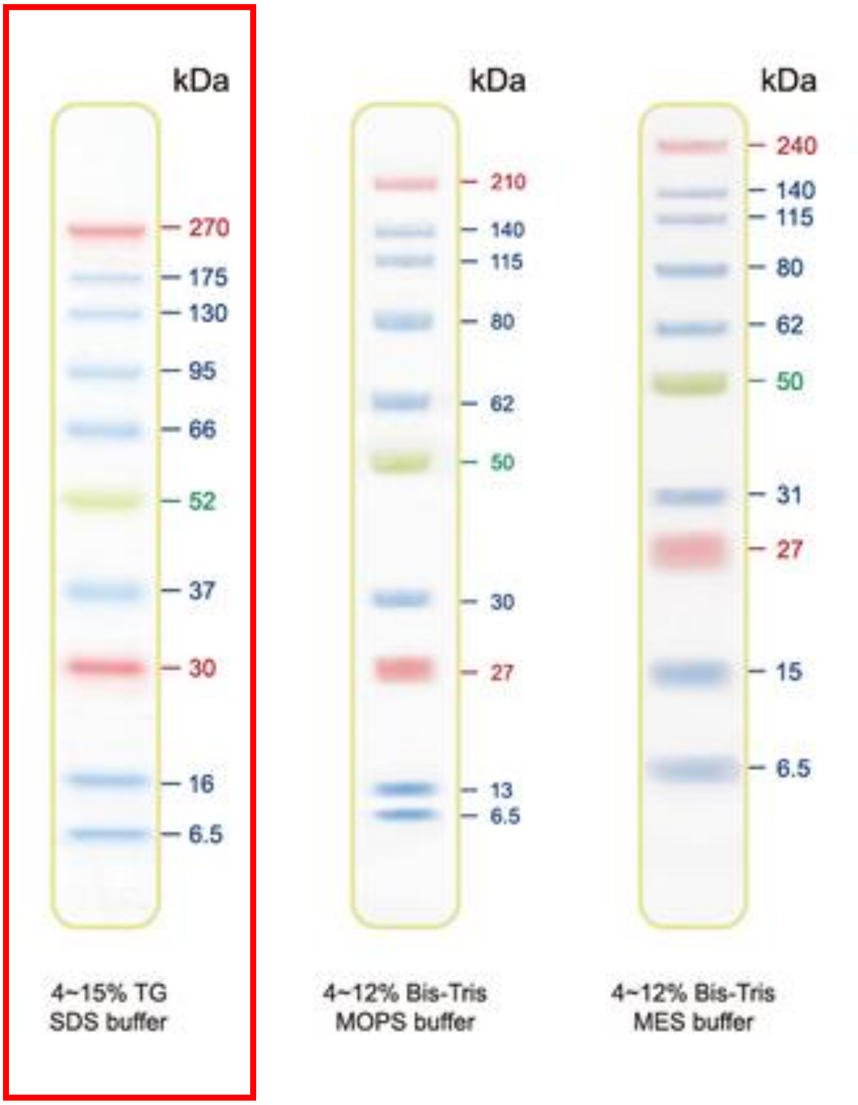

Standard protein ladder for western blot used Blu10 Plus (BLUltra) Prestained Protein Ladder (1BHC-PMB01-0500 BLUltra protein ladder, BIO-HELIX, Beining Rd, Keelung City, Taiwan)(M.W. from 6.5 to 270 kDa).

Supporting information

- **Nrf2**

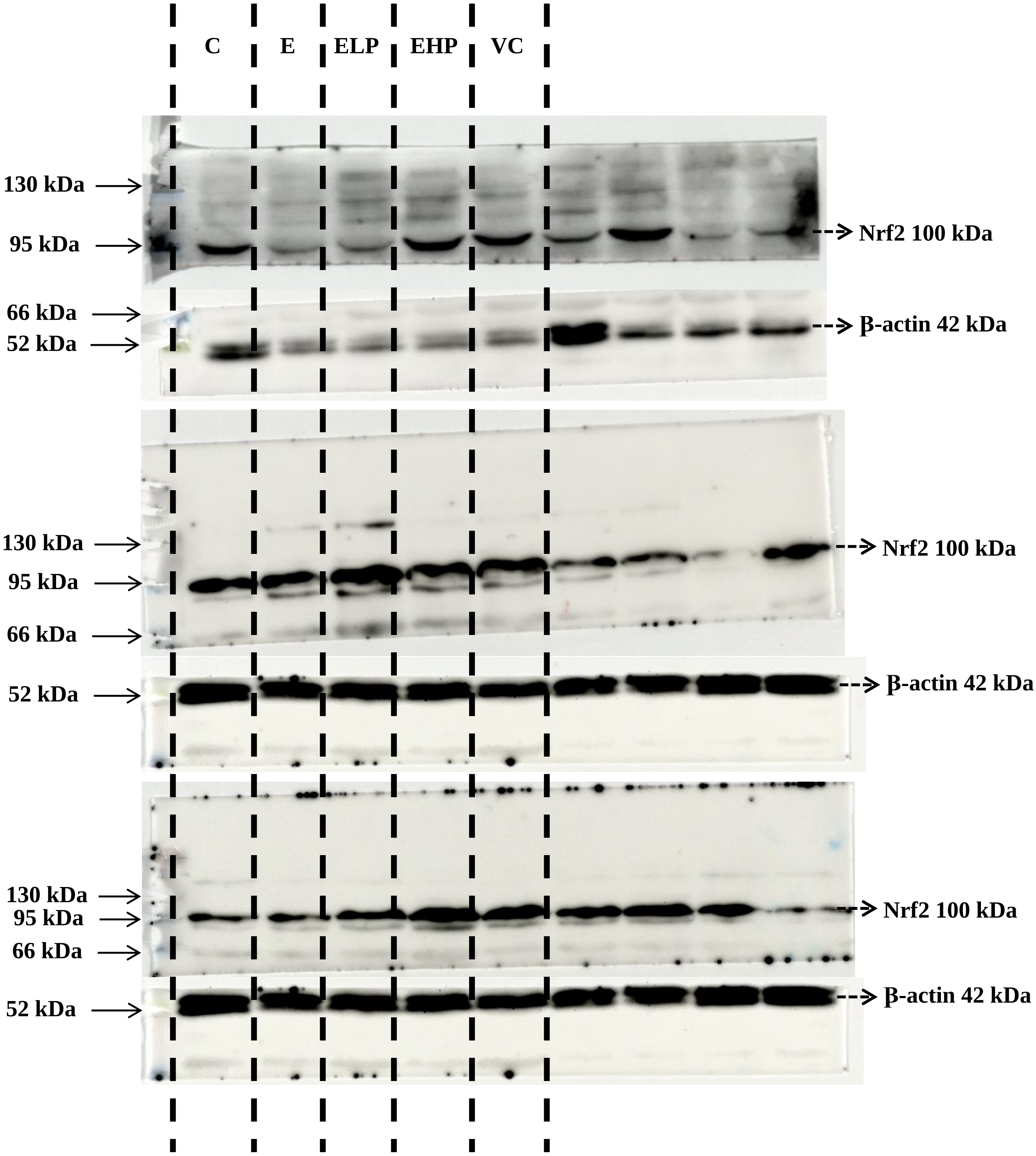

Supporting information

- **IRS-1**

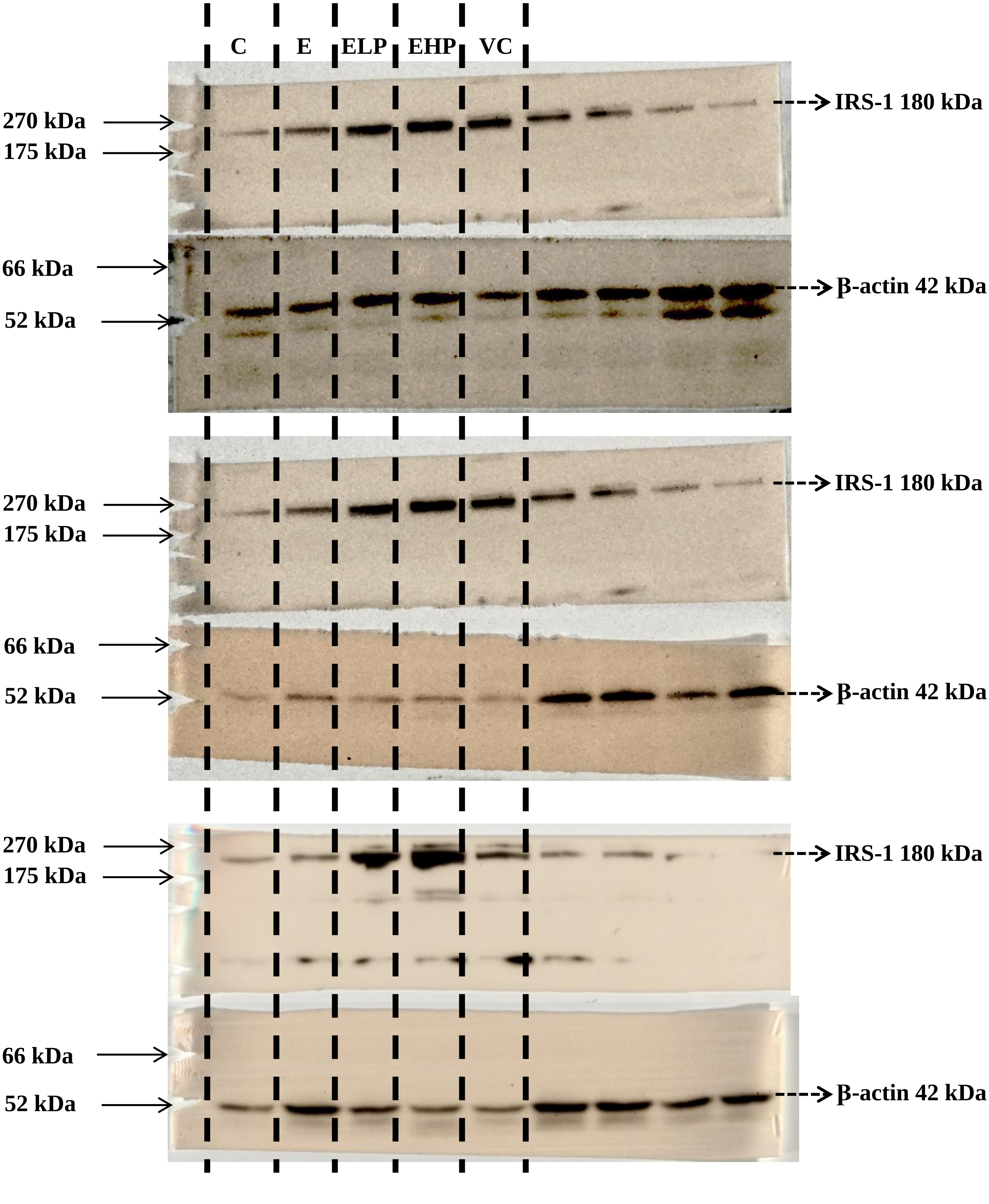

Supporting information

- **pAKT/AKT**

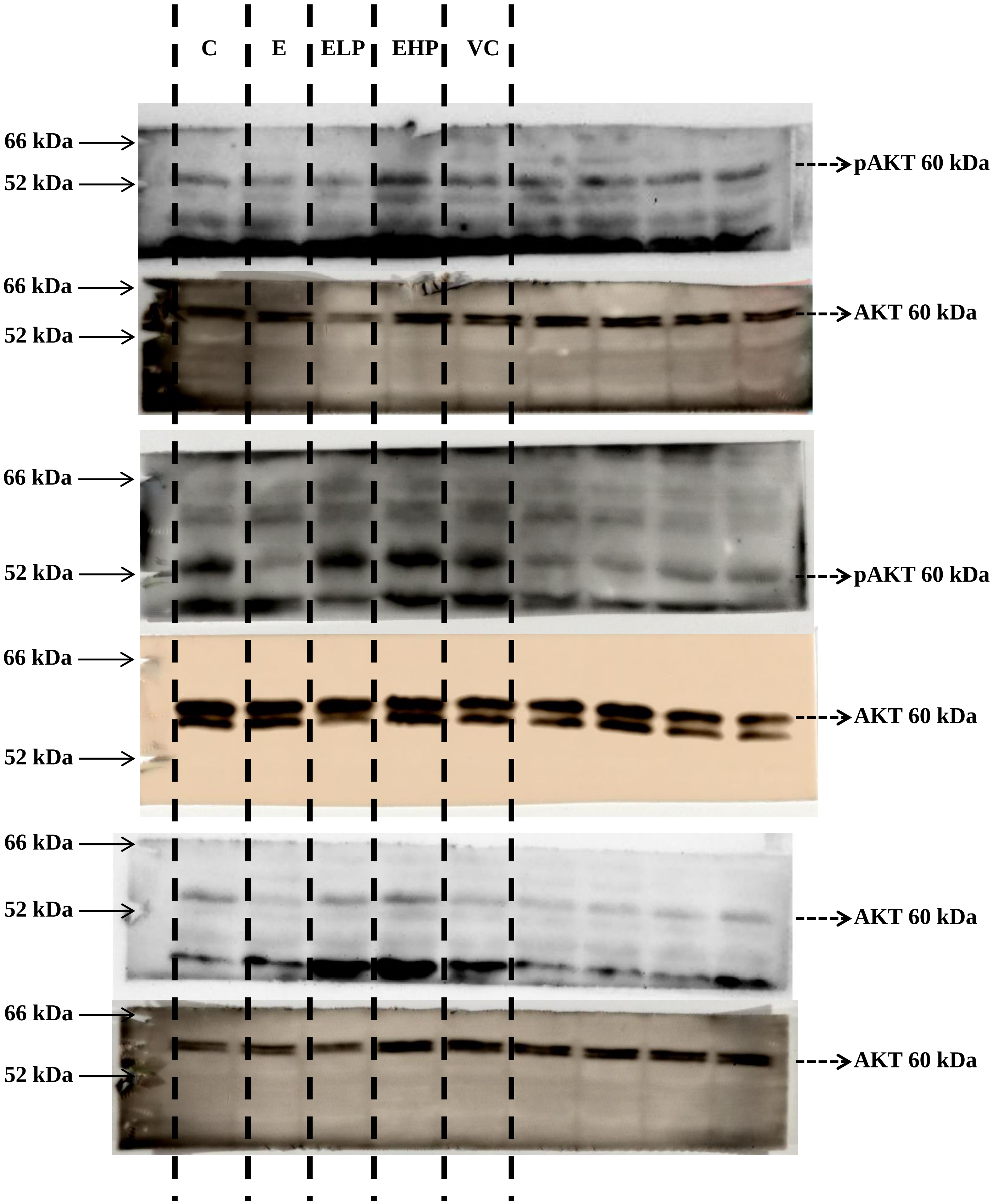

Supplement: S1 Raw images — (DOI: 10.5281/zenodo.12749098). (PDF) [file pone.0310138.s001.pdf]
