# Supplementary material for: Phycocyanin attenuates skeletal muscle damage and fatigue via modulation of Nrf2 and IRS-1/AKT/mTOR pathway in exercise-induced oxidative stress in rats
Source: PLoS One. 2024 Sep 10;19(9):e0310138. doi: 10.1371/journal.pone.0310138 (PMC11386417; doi:10.1371/journal.pone.0310138)
Supplement: Attachment — Submitted filename: Response to reviewer final.pdf [file pone.0310138.s003.pdf]

Comments to the Author

1. Is the manuscript technically sound, and do the data support the conclusions?
The manuscript must describe a technically sound piece of scientific research with data that supports the conclusions. Experiments must have been conducted rigorously, with appropriate controls, replication, and sample sizes. The conclusions must be drawn appropriately based on the data presented.

Reviewer #1: Yes

Reviewer #2: Yes

---

2. Has the statistical analysis been performed appropriately and rigorously?

Reviewer #1: No

Reviewer #2: I Don't Know

Response:

We apologize for the missing content regarding the statistical analysis. We have provided the statistical analysis detail under the material and method section in the revised MS.

---

3. Have the authors made all data underlying the findings in their manuscript fully available?
The PLOS Data policy requires authors to make all data underlying the findings described in their manuscript fully available without restriction, with rare exception (please refer to the Data Availability Statement in the manuscript PDF file). The data should be provided as part of the manuscript or its supporting information or deposited to a public repository. For example, in addition to summary statistics, the data points behind means, medians and variance measures should be available. If there are restrictions on publicly sharing data—e.g. participant privacy or use of data from a third party—those must be specified.

Reviewer #1: Yes

Reviewer #2: Yes

---

4. Is the manuscript presented in an intelligible fashion and written in standard English?
PLOS ONE does not copyedit accepted manuscripts, so the language in submitted articles must be clear, correct, and unambiguous. Any typographical or grammatical errors should be corrected at revision, so please note any specific errors here.

Reviewer #1: Yes

Reviewer #2: Yes

---

5. Review Comments to the Author
Please use the space provided to explain your answers to the questions above. You may also include additional comments for the author, including concerns about dual publication, research ethics, or publication ethics. (Please upload your review as an attachment if it exceeds 20,000 characters)

Reviewer #1: Study titled 'Phycocyanin attenuates skeletal muscle damage, atrophy, and fatigue via modulation of Nrf2, mTOR, MuRF-1, and Atrogin-1 in exercise-induced oxidative stress in rats.' It is indeed an interesting study. I have a few queries regarding the experiments and results.

Response:

We greatly appreciate the positive comment from the reviewer.

1. "How was Vitamin C specifically chosen as the positive control? The rationale of choosing this. For the exercise group in the context of the experiment focusing on muscle glucose uptake and glycogen content? The authors conducted experiments on two muscle types - the abdominal muscle and the soleus muscle as can be seen in the materials and method. But used only soleus. As noted, 'To assess muscle glucose uptake, the abdominal muscle was isolated (100–150 mg) and immersed in Krebs-Ringer bicarbonate (KRB) buffer [26]. Kindly clarify the same.

Response:

We are grateful that you find our research interesting. We chose vitamin C as the positive control since vitamin C or ascorbic acid is known to have high antioxidant activity, which protects the body from oxidative damage and helps strengthen the body's immunity [1]. Vitamin C is also used as a positive control in several studies related to exercise and oxidative stress [2,3] and is often used as a dietary supplement for athletes because of its strong antioxidant properties [4, 5]. Vitamin C reduces the oxidative stress effect on muscle fatigue, damage, and inflammation caused by exercise in skeletal muscles [4]. Previous studies have reported that vitamin C supplementation works effectively through the AKT/mTOR signaling pathway to reduce muscle atrophy [6], decreases muscle inflammation, muscle pain, and oxidative stress induced by acute exercise [7], and increases SR Ca^{2+} ATPase in the myocardium to improve myocardial contractility [8] and muscle tension [9], also increases glycogen content and glucose uptake activity in skeletal muscle through activated insulin signaling [10,11], which demonstrates a similar mechanism as phycocyanin.

For the concern regarding the muscle types, we apologize for unclear contents in the previous manuscript. We used two muscles, the soleus and quadriceps, both are muscle fiber type I [12,13]. Exercise clearly affects oxidative insulin-sensitive, glycolytic, and oxidative stress in the quadriceps muscle. Therefore, the quadriceps muscle is commonly used to test for muscle damage, glucose uptake, and insulin signaling [14,15]. The soleus muscle was used to determine the muscle force tension because of its small, and straight bundle shape. When electrically stimulated, clear changes can be seen according to the previous protocols [16-18]. We have added this information to the revised manuscript.

References

1. Ghalibaf MHE, Kianian F, Beigoli S, Behrouz S, Marefati N, Boskabady M, et al. The effects of vitamin C on respiratory, allergic and immunological diseases: an experimental and clinical-based review. Inflammopharmacology. 2023. doi:10.1007/s10787-023-01169-1
2. Close GL, Ashton T, Cable T, Doran D, Holloway C, McArdle F, et al. Ascorbic acid supplementation does not attenuate post-exercise muscle soreness following muscle-damaging exercise but may delay the recovery process. British Journal of Nutrition. 2006;95. doi:10.1079/bjn20061732
3. Yimcharoen M, Kittikunnathum S, Suknikorn C, Nak-On W, Yeethong P, Anthony TG, et al. Effects of ascorbic acid supplementation on oxidative stress markers in healthy women following a single bout of exercise. Journal of the International Society of Sports Nutrition. 2019;16. doi:10.1186/s12970-019-0269-8
4. Evans LW, Omaye ST. Use of saliva biomarkers to monitor efficacy of vitamin C in exercise-induced oxidative stress. Antioxidants. 2017. doi:10.3390/antiox6010005
5. Hemmatfar A, Piraki P, Sharif MAS, Behpour N. Evaluating the effect of Vitamin C on myocardial angiogenesis under oxidative stress induced by exhaustive exercise in rat. Pharmaceutical Sciences. 2018;24. doi:10.15171/PS.2018.40
6. Yang M, Teng S, Ma C, Yu Y, Wang P, Yi C. Ascorbic acid inhibits senescence in mesenchymal stem cells through ROS and AKT/mTOR signaling. Cytotechnology. 2018;70. doi:10.1007/s10616-018-0220-x

7. Righi NC, Schuch FB, De Nardi AT, Pippi CM, de Almeida Righi G, Puntel GO, et al. Effects of vitamin C on oxidative stress, inflammation, muscle soreness, and strength following acute exercise: meta-analyses of randomized clinical trials. European Journal of Nutrition. 2020. doi:10.1007/s00394-020-02215-2
8. Qin F, Yan C, Patel R, Liu W, Dong E. Vitamins C and E attenuate apoptosis, β-adrenergic receptor desensitization, and sarcoplasmic reticular Ca^{2+} ATPase downregulation after myocardial infarction. Free Radical Biology and Medicine. 2006;40. doi:10.1016/j.freeradbiomed.2006.01.019
9. Urso ML, Clarkson PM. Oxidative stress, exercise, and antioxidant supplementation. Toxicology. 2003. doi:10.1016/S0300-483X(03)00151-3
10. Bulduk E, Gönül B, Özer Ç. Effects of vitamin C on muscle glycogen and oxidative events in experimental diabetes. Molecular and Cellular Biochemistry. 2006;292. doi:10.1007/s11010-006-9226-3
11. Picklo MJ, Thyfault JP. Vitamin e and vitamin c do not reduce insulin sensitivity but inhibit mitochondrial protein expression in exercising obese rats. Applied Physiology, Nutrition and Metabolism. 2015;40. doi:10.1139/apnm-2014-0302
12. Kriketos AD, Pan DA, Sutton JR, Hoh JFY, Baur LA, Cooney GJ, et al. Relationships between muscle membrane lipids, fiber type, and enzyme activities in sedentary and exercised rats. American Journal of Physiology - Regulatory Integrative and Comparative Physiology. 1995;269. doi:10.1152/ajpregu.1995.269.5.r1154
13. Larson L, Lioy J, Johnson J, Medler S. Transitional Hybrid Skeletal Muscle Fibers in Rat Soleus Development. Journal of Histochemistry and Cytochemistry. 2019;67. doi:10.1369/0022155419876421
14. Yaspelkis BB, Singh MK, Trevino B, Krisan AD, Collins DE. Resistance training increases glucose uptake and transport in rat skeletal muscle. Acta Physiol Scand. 2002;175. doi:10.1046/j.1365-201X.2002.00998.x
15. Ezaki O, Higuchi M, Nakatsuka H, Kawanaka K, Itakura H. Exercise training increases glucose transporter content in skeletal muscles more efficiently from aged obese rats than young lean rats. Diabetes. 1992;41. doi:10.2337/diab.41.8.920
16. Park KH, Brotto L, Lehoang O, Brotto M, Ma J, Zhao X. Ex Vivo assessment of contractility, fatigability and alternans in isolated skeletal muscles. Journal of Visualized Experiments. 2012. doi:10.3791/4198
17. Vedsted P, Larsen AH, Madsen K, Sjøgaard G. Muscle performance following fatigue induced by isotonic and quasi-isometric contractions of rat extensor digitorum longus and soleus muscles in vitro. Acta Physiol Scand. 2003;178. doi:10.1046/j.1365-201X.2003.01123.x
18. Gomes ARS, Coutinho EL, França CN, Polonio J, Salvini TF. Effect of one stretch a week applied to the immobilized soleus muscle on rat muscle fiber morphology. Brazilian Journal of Medical and Biological Research. 2004;37. doi:10.1590/S0100-879X2004001000005

2. In the endpoint please mention, which muscles have been collected.

Response:

We apologize for the missing information. We have added details for the collected skeletal muscles under the material and method section. In brief, we used the soleus muscle for tension experiment, while the quadriceps were used in the remaining experiments.

3. Did the authors undertake an examination of fasting insulin levels in the animals as a means to evaluate insulin/glucose uptake? If so, it would be insightful to understand the baseline levels of both glucose and insulin.

Response:

Thank you for your suggestion. Previous studies reported that excessive exercise-induced oxidative stress decreases insulin sensitivity and glucose uptake activity

through decreased IRS-1 signaling [1,2], while nutrition supplementation in these conditions increases insulin sensitivity through increased IRS-1/PI3K/AKT signaling [3-5]. We therefore performed additional experiments to determine plasma insulin and glucose, and also the expression of the insulin receptor substrate-1 (IRS-1). Our study shows an increase in insulin sensitivity using QUICKI assessment [6] and increased glucose uptake activity through activation of the IRS-1 signaling pathway in excessive exercise combined with phycocyanin. These results helped us confirm insulin action in our study. We have added the new experiments and results in the revised MS.

References

1. Turgut M, Cinar V, Pala R, Tuzcu M, Orhan C, Telceken H, et al. Biotin and chromium histidinate improve glucose metabolism and proteins expression levels of IRS-1, PPAR-γ, and NF-KB in exercise-trained rats. Journal of the International Society of Sports Nutrition. 2018;15. doi:10.1186/s12970-018-0249-4
2. Chan SH, Kikkawa U, Matsuzaki H, Chen JH, Chang WC. Insulin receptor substrate-1 prevents autophagy-dependent cell death caused by oxidative stress in mouse NIH/3T3 cells. Journal of Biomedical Science. 2012;19. doi:10.1186/1423-0127-19-64
3. Wang H, Wang J, Zhu Y, Yan H, Lu Y. Effects of different intensity exercise on glucose metabolism and hepatic IRS/PI3K/AKT pathway in sd rats exposed with TCDD. International Journal of Environmental Research and Public Health. 2021;18. doi:10.3390/ijerph182413141
4. Kerksick CM, Wilborn CD, Roberts MD, Smith-Ryan A, Kleiner SM, Jäger R, et al. ISSN exercise & sports nutrition review update: Research & recommendations. Journal of the International Society of Sports Nutrition. 2018. doi:10.1186/s12970-018-0242-y
5. Turgut M, Cinar V, Pala R, Tuzcu M, Orhan C, Telceken H, et al. Biotin and chromium histidinate improve glucose metabolism and proteins expression levels of IRS-1, PPAR-γ, and NF-KB in exercise-trained rats. Journal of the International Society of Sports Nutrition. 2018;15. doi:10.1186/s12970-018-0249-4
6. Quon MJ. QUICKI Is a Useful and Accurate Index of Insulin Sensitivity. Journal of Clinical Endocrinology & Metabolism. 2002;87. doi:10.1210/jc.87.2.949

4. In the analysis of NRf2 blots, an intriguing observation is the disparity between the representation in Vitamin C graphs and the actual expression in the same lane. To gain a comprehensive understanding, could the authors provide details on how many times the gel was run? Additionally, whether authors used soleus muscles were pooled or analyzed individually from each animal?

Response:

Thank you for your comment. We have performed an additional Nrf2 western blotting and corrected the inconsistency between the NRf2 expression and histogram. By choosing the western blot image that represents the histogram as shown in Fig.5.

We have also added additional detail on the western blot as suggested. The quadriceps were used to determine the expression of proteins of interest. We used 3 individual muscle samples/groups.

5. Given the nature of the soleus muscle as a slow-fiber muscle, it would be good if authors conduct fiber typing, preferably using techniques like COX/DSH staining or ATPase staining. This would enhance the robustness of the conclusion that phycocyanin contributes to an improvement in the observed conditions.

Response:

We appreciate your comment. We decided to perform an additional experiment to determine whether phycocyanin has any effect on ATP or not. We used the real-time PCR technique to study the gene expression of ATP synthase (*Mitochondrial ATP synthase F1 subunit beta* (*ATP5B*)). Although, this is not to confirm the muscle type, but it can provide an insightful mechanism of the PC extraction on energy expenditure by muscle cells. A previous study reported that decreased ATPase, ATP synthase, and ATP content lead to decreased ATP synthesis in excessive exercise [1,2]. Our additional result showed that the *ATP5B* was activated which implies that phycocyanin can stimulate the ATP synthase. This is required for ATP production during exercise. The finding contributes to the beneficial effect of phycocyanin to improve the condition of exercise-induced oxidative stress.

References

1. Tarini VAF, Carnevali LC, Arida RM, Cunha CA, Alves ES, Seeleander MCL, et al. Effect of exhaustive ultra-endurance exercise in muscular Glycogen and both Alpha1 and Alpha2 AMPK protein expression in trained rats. Journal of Physiology and Biochemistry. 2013;69. doi:10.1007/s13105-012-0224-5
2. Schluter JM, Fitts RH. Shortening velocity and ATPase activity of rat skeletal muscle fibers: Effects of endurance exercise training. American Journal of Physiology-Cell Physiology. 1994;266. doi:10.1152/ajpcell.1994.266.6.c1699

6. What could be the possible reasons for the increase in LDH during exercise? While there is no change in antioxidant parameters when comparing exercise and control conditions. How do the authors interpret this finding?

Response:

Thank you for your question. Lactate dehydrogenase (LDH) is an enzyme that catalyzes the exchange between pyruvate and lactate in cells. The membrane lipid bilayer prevents LDH from leaking out to the extracellular compartment. However, exercise-induced oxidative stress results in membrane lipid peroxidation at the membrane lipid bilayer, leading to membrane dysfunction.

Therefore, the leakage of LDH can be observed in the exercise group as shown in our study. LDH is a marker used to assess tissue damage or injury [1-3].

A previous study showed that decreased or no change in antioxidant activity due to excessive exercise-induced oxidative stress, while increased MDA was observed in excessive exercise than non-exercise [4]. These reports are similar to our study to show that antioxidants do not change in the exercise group, but an elevated lipid peroxidation was significantly determined when compared with the other groups (C, ELP, EHP, and VC groups). Therefore, increased lipid peroxidation in the excessive exercise results in an increased leaking of LDH due to lipid membrane dysfunction.

References

1. Olayeriju OS, Olaleye MT, Crown OO, Komolafe K, Boligon AA, Athayde ML, et al. Ethylacetate extract of red onion (Alliumcepa L.) tunic affects hemodynamic parameters in rats. Food Science and Human Wellness. 2015;4. doi:10.1016/j.fshw.2015.07.002
2. Tie S, Zhang L, Li B, Xing S, Wang H, Chen Y, et al. Effect of dual targeting procyanidins nanoparticles on metabolomics of lipopolysaccharide-stimulated inflammatory macrophages. Food Science and Human Wellness. 2023;12. doi:10.1016/j.fshw.2023.03.045
3. Papadopoulos NM, Leon AS, Bloor CM. Effects of Exercise on Plasma and Tissue Levels of Lactate Dehydrogenase and Isoenzymes in Rats. Proceedings of the Society for Experimental Biology and Medicine. 1967;125. doi:10.3181/00379727-125-32260
4. Wang Y, Xiang Y, Wang R, Li X, Wang J, Yu S, et al. Sulforaphane enhances Nrf2-mediated antioxidant responses of skeletal muscle induced by exhaustive exercise in HIIT mice. Food Science and Human Wellness. 2022;11. doi:10.1016/j.fshw.2022.04.035

7. However, the tension study with phycocyanin is inconclusive, and further studies may be needed to better understand its mechanism on muscle tension. Could the author provide insights into the type of study that would be instrumental in proving the mechanism?

Response:

Thank you for your question. Since muscle contraction requires ATP and Ca^{2+} [1], if we could perform a future experiment, we would study the ryanodine receptor type 1 (RYR1). This is because RYR1 serves as the skeletal muscle calcium release channel [2]. If the expression of RYR1 increases, it indicates increased calcium release in the skeletal muscles. An optional experiment would be the study of the Ca^{2+} concentration. If Ca^{2+} is released in greater quantities, it leads to increased muscle contraction [3].

References

1. Adams RJ, Schwartz A. Comparative mechanisms for contraction of cardiac and skeletal muscle. Chest. 1980. doi:10.1378/chest.78.1.123

2. Lawal TA, Todd JJ, Meilleur KG. Ryanodine Receptor 1-Related Myopathies: Diagnostic and Therapeutic Approaches. Neurotherapeutics. 2018. doi:10.1007/s13311-018-00677-1
3. Martins AS, Shkryl VM, Nowycky MC, Shirokova N. Reactive oxygen species contribute to Ca^{2+} signals produced by osmotic stress in mouse skeletal muscle fibres. Journal of Physiology. 2008;586. doi:10.1113/jphysiol.2007.146571

8. Additionally, in the table, the authors mentioned Quadriceps muscle weight (g) (MW), but all experiments utilized the soleus muscle, and the soleus muscle weight is provided. This inconsistency in muscle type raises questions about the accuracy of the reported data.

Response:

We apologize for causing confusion. We used two muscles in the present study, including the soleus and the quadriceps. All muscles are classified as slow-twitch or muscle fiber type I [1,2]. We have provided additional information in the revised MS. In summary, the soleus was used to measure muscle force tension, and the quadriceps were used in muscle glucose uptake, western blotting, real-time PCR, and biomarkers of muscle damage and antioxidants assay. We have added the muscle weight of both muscles in the revised MS.

References

1. Kriketos AD, Pan DA, Sutton JR, Hoh JFY, Baur LA, Cooney GJ, et al. Relationships between muscle membrane lipids, fiber type, and enzyme activities in sedentary and exercised rats. American journal of physiology. Regulatory, integrative and comparative physiology. 1995;269. doi:10.1152/ajpregu.1995.269.5.r1154
2. Larson L, Lioy J, Johnson J, Medler S. Transitional Hybrid Skeletal Muscle Fibers in Rat Soleus Development. Journal of Histochemistry and Cytochemistry. 2019;67. doi:10.1369/0022155419876421

9. An intriguing observation is the maintenance of protein balance. Have the authors explored the pAKT/IGF signaling pathway or referred to any existing reports demonstrating that Phycocyanin activates the AKT/IGF signaling pathway?

Response:

We greatly appreciate your suggestion. We have carried out additional experiments to determine the pAKT and AKT pathway as suggested. AKT signaling can alter protein metabolism including protein synthesis, protein degradation, and glucose absorption in skeletal muscle. The additional result demonstrated the decreased pAKT/AKT expression in the E group, while the treatment group with phycocyanin shows the activation of pAKT/AKT expression that lead to increased glucose uptake and reduced muscle atrophy. The new contents were added in the revised MS.

10. Upon scrutiny, the histology picture presented in the panel raises concerns about the visibility of myofibers, particularly noting poor staining in panels ELP and EHP. Could the authors revise/clear on the methodology employed to calculate fiber numbers and provide a clear definition of fiber number? Furthermore, it would be valuable to include measurements of fiber diameter.

Response:

We apologize for the unclear histology picture and are grateful for the guidance on diameters. We have added the result of fiber diameter as suggested.

Regarding the fiber number, we fixed the staining in the ELP and EHP panels and added the new histological images in Fig. 7. The fiber number was calculated using the count of muscle fibers with clear boundaries as shown in the provided image below, and data analyzed using Image Processing and Analysis in Java (ImageJ).

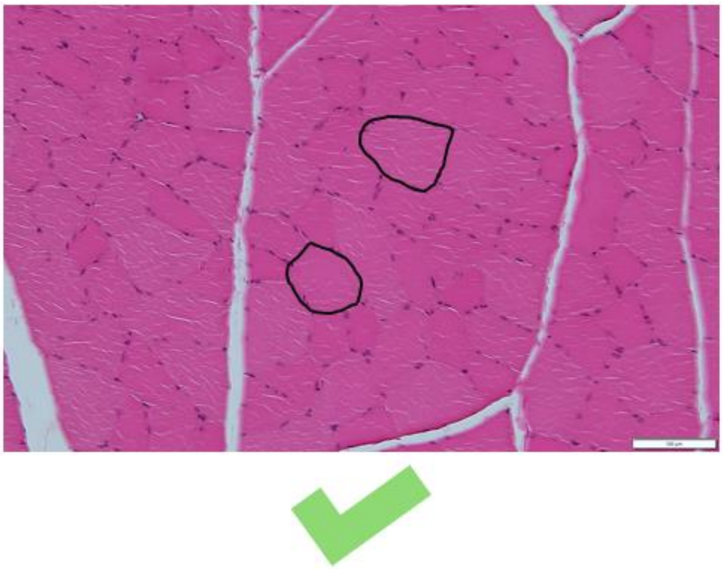

11. Regarding the statistical analysis description, there is an apparent complexity that may hinder comprehension. A thorough revision is recommended to ensure a clear and detailed explanation for better understanding.

Response:

We apologize for the missing information. We have added the statistical explanations under the statistical analysis section.

Reviewer #2: Dear writers,
This report describes the effect of Phycocyanin against exercise-induced oxidative stress in a rat model. The results are interesting and this report seems suitable for publication in PLOS ONE. However, it should be noted that the final decision will be made by the editor. Additionally, there are some issues that need to be fixed before the article is published.

Response:

We greatly appreciate the positive response from the reviewer.

The following issues need to be reviewed.

1. Statistical analysis is not specified in the material-method section. Statistical explanations are briefly included only in the description of tables 1 and 2, however, it is not stated how the other data were evaluated statistically.

Response:

We apologize for the missing detail on statistical analysis in the method. We have added the statistical explanations under the statistical analysis section.

2. It was shown that both body weight and quadriceps muscle weight decreased significantly in the EHP group (table 1). This result is not discussed in the manuscript. Additionally, contradictory to these data, it was stated that phycocyanin increased protein synthesis and decreased protein degradation (lines 283, 284, 285).

Response:

Thank you for this interesting question. As shown in table 1, the body weight and quadriceps muscle weight decreased in the EHP group. Exercise increases metabolism, affecting decreased body weight and body composition [1]. Previous studies have shown that the effect of phycocyanin improves metabolism in diabetic rats [2] and decreases body weight in obese mice [3].

Our study shows that the EHP significantly decreased the quadriceps muscle weight and body weight compared to the C and E groups. The reduced quadriceps and body weight in the EHP group may be due to an increased high metabolism on exercise combined with phycocyanin.

Muscle weight is the total weight of the muscle, while muscle mass index is muscle weight divided by body weight. Therefore, the muscle mass index represents the ratio of muscle to body weight.

Even the reduced quadriceps weight was observed but there was no significant decrease in the muscle mass index (MW/BW) when compared with the other groups. This indicates that phycocyanin did not affect the reduced muscle mass.

In addition, the previous studies reported that muscle atrophy rats presented decreased muscle fiber cross-sectional area and muscle fiber diameter or muscle fiber size, although there were decreases or no changes in muscle mass [4-6]. This is consistent with our study, the E group shows a decreased muscle fiber size than the C group, and phycocyanin supplementation shows an increased muscle fiber size than the E group.

In conclusion, the quadriceps muscle weight decreased from a decrease in body weight, but muscle mass did not decrease. These results do not conflict with the results of phycocyanin increased protein synthesis and decreased protein degradation. To confirm using H&E staining, the result shows an increase in the muscle fiber cross-sectional area and muscle fiber diameter in the EHP groups than in the E group.

We have already added this concern in the discussion section of the revised MS.

References

1. Caton SJ, Bielohuby M, Bai Y, Spangler LJ, Burget L, Pfluger P, et al. Low-carbohydrate high-fat diets in combination with daily exercise in rats: Effects on body weight regulation, body composition and exercise capacity. Physiol Behav. 2012;106. doi:10.1016/j.physbeh.2012.02.003

2. Husain A, Alouffi S, Khanam A, Akasha R, Farooqui A, Ahmad S. Therapeutic Efficacy of Natural Product 'C-Phycocyanin' in Alleviating Streptozotocin-Induced Diabetes via the Inhibition of Glycation Reaction in Rats. Int J Mol Sci. 2022;23. doi:10.3390/ijms232214235

3. Silva-Neto AF, Fratelli C, Pucci VG, Boldarine VT, Ferreira YAM, Telles MM, et al. C-phycocyanin extracted from Spirulina using a green solvent approach presents an anti-obesity characteristic in mice fed a hyperlipidic diet. J Funct Foods. 2023;108. doi:10.1016/j.jff.2023.105747

4. Naito H, Powers SK, Demirel HA, Sugiura T, Dodd SL, Aoki J. Heat stress attenuates skeletal muscle atrophy in hindlimb-unweighted rats. Journal of Applied Physiology. 2000;88. doi:10.1152/jappl.2000.88.1.359

5. Fan Z, Xiao Q. Impaired autophagic flux contributes to muscle atrophy in obesity by affecting muscle degradation and regeneration. Biochemical and Biophysical Research Communications. 2020;525. doi:10.1016/j.bbrc.2020.02.110

6. Hasan MM, Shalaby SM, El-Gendy J, Abdelghany EMA. Beneficial effects of metformin on muscle atrophy induced by obesity in rats. The Journal of Cellular Biochemistry. 2019;120. doi:10.1002/jcb.27852

3. There are abbreviation errors throughout the text. Abbreviations should be explained in full when first used in the text and then only abbreviations should be used.

Response:

Thank you for the concern. We have corrected this issue throughout the manuscript.

4. References need to be reviewed. For example, in the 4th reference, the authors' titles are written. Volume and page numbers of some references are not written (e.g. 34, 35).

Response:

Thank you for your suggestion. We have revised and corrected the reference parts.

---

6. PLOS authors have the option to publish the peer review history of their article (what does this mean?). If published, this will include your full peer review and any attached files.

If you choose "no", your identity will remain anonymous but your review may still be made public.

Do you want your identity to be public for this peer review? For information about this choice, including consent withdrawal, please see our Privacy Policy.

Reviewer #1: No

Reviewer #2: No
