# Supplementary material for: Phycocyanin attenuates skeletal muscle damage and fatigue via modulation of Nrf2 and IRS-1/AKT/mTOR pathway in exercise-induced oxidative stress in rats
Source: PLoS One. 2024 Sep 10;19(9):e0310138. doi: 10.1371/journal.pone.0310138 (PMC11386417; doi:10.1371/journal.pone.0310138)
Supplement: Attachment — Submitted filename: comments PLOS.docx [file pone.0310138.s002.docx]

Study titled 'Phycocyanin attenuates skeletal muscle damage, atrophy, and fatigue via modulation of Nrf2, mTOR, MuRF-1, and Atrogin-1 in exercise-induced oxidative stress in rats.' It is indeed an interesting study. I have a few queries regarding the experiments and results.

1. "How was Vitamin C specifically chosen as the positive control? The rationale of choosing this. For the exercise group in the context of the experiment focusing on muscle glucose uptake and glycogen content? The authors conducted experiments on two muscle types - the abdominal muscle and the soleus muscle as can be seen in the materials and method. But used only soleus. As noted, 'To assess muscle glucose uptake, the abdominal muscle was isolated (100–150 mg) and immersed in Krebs-Ringer bicarbonate (KRB) buffer [26]. Kindly clarify the same.
2. In the endpoint please mention, which muscles have been collected.
3. Did the authors undertake an examination of fasting insulin levels in the animals as a means to evaluate insulin/glucose uptake? If so, it would be insightful to understand the baseline levels of both glucose and insulin.
4. In the analysis of NRf2 blots, an intriguing observation is the disparity between the representation in Vitamin C graphs and the actual expression in the same lane. To gain a comprehensive understanding, could the authors provide details on how many times the gel was run? Additionally, whether authors used soleus muscles were pooled or analyzed individually from each animal?
5. Given the nature of the soleus muscle as a slow-fiber muscle, it would be good if authors conduct fiber typing, preferably using techniques like COX/DSH staining or ATPase staining. This would enhance the robustness of the conclusion that phycocyanin contributes to an improvement in the observed conditions.
6. What could be the possible reasons for the increase in LDH during exercise? While there is no change in antioxidant parameters when comparing exercise and control conditions. How do the authors interpret this finding?
7. However, the tension study with phycocyanin is inconclusive, and further studies may be needed to better understand its mechanism on muscle tension. Could the author provide insights into the type of study that would be instrumental in proving the mechanism?
8. Additionally, in the table, the authors mentioned Quadriceps muscle weight (g) (MW), but all experiments utilized the soleus muscle, and the soleus muscle weight is provided. This inconsistency in muscle type raises questions about the accuracy of the reported data.
9. An intriguing observation is the maintenance of protein balance. Have the authors explored the pAKT/IGF signaling pathway or referred to any existing reports demonstrating that Phycocyanin activates the AKT/IGF signaling pathway?
10. Upon scrutiny, the histology picture presented in the panel raises concerns about the visibility of myofibers, particularly noting poor staining in panels ELP and EHP. Could the authors revise/clear on the methodology employed to calculate fiber numbers and provide a clear definition of fiber number? Furthermore, it would be valuable to include measurements of fiber diameter.
11. Regarding the statistical analysis description, there is an apparent complexity that may hinder comprehension. A thorough revision is recommended to ensure a clear and detailed explanation for better understanding.
